# Pituitary MRI, Clinical and Biochemical Findings in Children With Growth Disorder: A Retrospective Comparative Study

**DOI:** 10.7759/cureus.112339

**Published:** 2026-07-09

**Authors:** Moneera K Mohamed, Ahmed Ibrahim, Yassin Alsaleh, Manal Alshawi, Isaq Almughaziel, Wail Medani, Abdulhameed A Al-Bunyan

**Affiliations:** 1 Pediatric Endocrinology, Maternity and Children Hospital, Al Ahsa, SAU; 2 Public Health, College of Applied Medical Sciences, King Faisal University, Al Hofuf, SAU; 3 Occupational Health, Prince Sultan Cardiac Center, Al Ahsa, SAU; 4 Pediatric DM and Endocrine Units, Maternity and Children Hospital, Al Ahsa, SAU

**Keywords:** children, deficiency, growth hormone, idiopathic short stature, isolated, multiple, pituitary

## Abstract

Background

Growth disorders in children have substantial consequences for physical development and long-term health. This study assesses pituitary gland MRI, clinical, and biochemical laboratory findings in children with growth disorders.

Methods

A retrospective analytical study was conducted at the Maternity and Children Hospital, Al-Ahsa, Saudi Arabia, including 267 children diagnosed with isolated growth hormone deficiency (IGHD), multiple pituitary hormone deficiency (MPHD), or idiopathic short stature (ISS). Demographic, anthropometric, laboratory, and MRI findings were analyzed.

Results

Males constituted 155 (58.1%) of the cohort. Most children were born via vaginal cephalic delivery (233, 87.3%), with no significant sex differences in birth presentation or birth weight, indicating minimal perinatal contribution to growth abnormalities. Body mass index (BMI) differed significantly between sexes (*P* < 0.0001). Male children were more likely to be overweight, whereas underweight was slightly more prevalent among females. Furthermore, females presented and received a diagnosis at significantly younger ages than males, with mean ages at presentation of 100.4 ± 35.5 versus 114.6 ± 33.1 months (P < 0.0009) and mean ages at diagnosis of 108.2 ± 34.6 versus 123.0 ± 33.9 months (P = 0.0005), respectively. IGHD was the most prevalent diagnosis (222, 83.1%), followed by ISS (42, 15.7%) and MPHD (3, 1.1%), with significant sex variation across diagnoses (P < 0.01). Baseline growth velocity differed significantly, being lowest in MPHD (1.7 ± 1.5 cm/year), followed by IGHD (3.3 ± 1.01 cm/year) and ISS (3.6 ± 1.1 cm/year) (P < 0.001); however, growth velocity normalized across groups after two years of growth hormone therapy (P = 0.9). Pituitary MRI findings showed a normal brain, and the pituitary gland was the most frequent MRI finding, observed in 174 children (65.2%). Among these patients, the majority were diagnosed with IGHD (139 cases, 69.9%), followed by ISS (33 cases, 19.0%), whereas only two cases (1.1%) had MPHD. This finding suggests that a substantial proportion of children with growth disorders, particularly those with IGHD, may not exhibit detectable structural abnormalities on MRI, emphasizing the functional rather than anatomical nature of many growth hormone deficiencies.

Conclusion

The findings of this study suggest that growth disorders in children are predominantly characterized by IGHD, are influenced more by endocrine dysfunction than by perinatal factors or overt structural brain abnormalities, and respond favorably to growth hormone therapy, which effectively normalizes growth velocity over time. These results emphasize the importance of early diagnosis, comprehensive endocrine assessment, and targeted imaging evaluation to optimize clinical outcomes in children with impaired growth.

## Introduction

Growth hormone deficiency (GHD) is an important endocrine disorder in children. The cause and mechanism of GHD may be congenital, acquired or idiopathic. There is variation in the cause of GH deficiency, which can be isolated or part of multiple pituitary hormone deficiency [1-3]. Current evidence indicates that effective evaluation and management of growth disorders require standardized growth assessment, appropriate endocrine investigations, population-specific growth references, and multidisciplinary diagnostic approaches to optimize growth outcomes and long-term health [4-6].

Although these conditions share short stature as a common phenotype, differences exist in clinical presentation, biochemical profiles, MRI findings, and response to therapy. Early diagnosis and treatment with recombinant GH are essential to optimize height outcomes. To our knowledge, no local comparative studies have analyzed growth patterns, hormonal profiles, and radiological abnormalities among isolated growth hormone deficiency (IGHD), multiple pituitary hormone deficiency (MPHD), and idiopathic short stature (ISS) in children. Determinants of differential response and long-term outcomes are scarce in the study population.

This study provided a comprehensive analytic assessment of children with growth disorders and identified some predictors of growth response and some clinical management outcomes.

This study evaluated the associations between diagnostic categories of growth disorders, perinatal characteristics, growth outcomes, and magnetic resonance imaging (MRI) findings among children diagnosed with isolated growth hormone deficiency (IGHD), multiple pituitary hormone deficiency (MPHD), and idiopathic short stature (ISS). The findings provide important insights into the clinical, auxological, and radiological characteristics of these disorders and are consistent with several previous reports.

## Materials and methods

Study setting and design

This retrospective analytical study was conducted at the Pediatric Department of the Maternity and Children Hospital (MCH) in Al-Ahsa, Saudi Arabia. The study aimed to evaluate the clinical, laboratory, radiological, and growth characteristics of children diagnosed with growth disorders and followed at the institution.

Study population and sampling

The study population included children born at the Maternity and Children Hospital (MCH), Al-Ahsa, who underwent evaluation for short stature and were subsequently diagnosed with isolated growth hormone deficiency (IGHD), multiple pituitary hormone deficiency (MPHD), or idiopathic short stature (ISS). A total population sampling approach was employed, whereby all eligible patients diagnosed between 1 January 2010 and 30 June 2025 were included in the study.

Inclusion and exclusion criteria

Children aged ≤14 years at the time of diagnosis with confirmed growth hormone deficiency based on growth hormone stimulation testing and those of the same age diagnosed with idiopathic short stature after exclusion of other identifiable causes of short stature were included.

Children were excluded if they were >14 years at diagnosis or if they had chronic medical conditions attributed to their short stature.

Data sources and collection

Data were collected retrospectively from hospital medical records, laboratory databases, magnetic resonance imaging (MRI) reports, and follow-up growth charts. Relevant demographic, clinical, laboratory, radiological, and treatment-related information was extracted using a standardized data collection form.

Study variables

The collected variables included: (A) Demographic characteristics: age, sex, birth weight, gestational age, and mode of delivery. (B) Anthropometric measurements: height, weight, and body mass index (BMI). (C) Magnetic resonance images (MRI) findings of the brain, pituitary gland, and hypothalamic region. (D) Growth outcomes: annual growth velocity and catch-up growth after one and two years of growth hormone therapy. (E) Laboratory investigations: peak growth hormone levels during stimulation testing, pituitary and thyroid function tests.

Statistical analysis

Data were analyzed using IBM Statistical Package for the Social Sciences (SPSS), Version 30 (IBM Corp., Armonk, NY, USA). Descriptive statistics were used to summarize baseline characteristics, with continuous variables presented as means ± standard deviations (SD), and categorical variables expressed as frequencies and percentages. Associations between categorical variables were assessed using the Chi-square test, while differences in continuous variables between study groups were evaluated using the Student’s t-test and One-way analysis of variance (ANOVA) with F-statistics were used to compare the mean values of continuous variables across the three diagnostic groups. Statistical significance was determined at a 95% confidence level, and a p-value < 0.05 was considered statistically significant.

## Results

Basic characteristics of study participants

Table 1 summarizes the sociodemographic and clinical characteristics of 267 children with growth disorder included in the study, including 112 females (41.9%) and 155 males (58.1%). There was no statistically significant difference in birth presentation between sexes (χ² = 3.23, P = 0.36). Overall, the majority of children were delivered by vaginal cephalic presentation (87.3%), followed by cesarean section with cephalic presentation (10.1%), whereas cesarean section breech (1.9%) and vaginal breech (0.7%) deliveries were uncommon. Similar distributions were observed among females and males.

**Table 1:** Basic characteristics of study participants (n=267) * : Chi-squared test (χ²) ** : T-test IGHD: Isolated Growth Hormone Deficiency; MPHD: Multiple Pituitary Hormone Deficiency; ISS: Idiopathic Short Stature.

Variable	Total (N=267)	Female (112, 41.9%)	Male (155, 58.1%)	Test Statistic	P value
Birth Presentation					
Cesarean Section breech	5(1.9)	2(07)	3(1.1)	3.23*	0.36
Cesarean Section Cephalic	27(10.1%)	7(2.6%)	20(7.5%)		
Vaginal breech	2(0.7%)	1(0.4%)	1(0.4%)		
Vaginal cephalic	233(87.3%)	102(38.2%)	131(49.1%)		
Birth weight (kg)					
<2.5	38(12.2%)	16(6.0%)	22(8.2%)	0.0005*	0.98
≥2.5	229(85.5%)	96(36.0%)	133(49.8%)		
BMI (kg/m^2^)					
> 85^th^ percentile	20(7.5%)	5(1.9%)	15(5.6%)	2.69*	0.26
>5^th^ - ≤85^th^ percentile	170(63.7%)	75(28.1%)	95(35.6%)		
≤5^th^	77(28.8%)	32(12.0%)	45(16.9%)		
Diagnosis					
IGHD	222(83.1%)	84(31.5%)	138(51.7%)	9.16*	0.01
MPHD	3(1.1%)	2(0.7%)	1(0.4%)		
ISS	42(15.7%)	26(9.7%)	16(6.0%)		
Age at diagnosis (mean±SD) months	116.2±35.2	108.2±34.6	123.0±33.9	3.56**	0.0005
Age at presentation (mean±SD) months	108.3±35	100.4±35.5	114.6±33.1	3.35**	0.0009

There was no significant difference in birth weight distribution between sexes (χ² = 0.0005, P = 0.98). Most children (85.5%) had a birth weight of ≥2.5 kg, including 96 females (36.0%) and 133 males (49.8%), whereas only 38 children (12.2%) had a birth weight below 2.5 kg.

Body mass index (BMI) categories did not differ significantly between females and males (χ² = 2.69, P = 0.26). The majority of participants had a BMI between the 5th and 85th percentiles (63.7%), while 28.8% had a BMI below the 5th percentile and 7.5% had a BMI above the 85th percentile. Although overweight status (BMI >85th percentile) was more frequent among males (5.6%) than females (1.9%), and underweight status (BMI <5th percentile) was slightly more common among males (16.9%) than females (12.0%), these differences were not statistically significant.

A significant sex-related difference was observed in the distribution of diagnoses (χ² = 9.16, P = 0.01). Isolated growth hormone deficiency (IGHD) was the most common diagnosis, affecting 222 children (83.1%), including 84 females (31.5%) and 138 males (51.7%). Idiopathic short stature (ISS) was identified in 42 children (15.7%), with a higher proportion among females than males (9.7% vs. 6.0%). Multiple pituitary hormone deficiency (MPHD) was rare, occurring in only three children (1.1%), including two females and one male.

Furthermore, females presented for clinical evaluation and received a diagnosis at significantly younger ages than males. The mean age at presentation was 100.4 ± 35.5 months among females compared with 114.6 ± 33.1 months among males (t = 3.35, P = 0.0009). Similarly, the mean age at diagnosis was significantly lower in females than in males (108.2 ± 34.6 vs. 123.0 ± 33.9 months, respectively; t = 3.56, P = 0.0005). These findings indicate that, despite similar birth characteristics and BMI distributions, females were evaluated and diagnosed earlier than males, and the distribution of growth-related diagnoses differed significantly between the sexes.

Association between the diagnosis, birth presentation, sex and birth weight

Table 2 shows the association between the diagnosis, birth presentation, sex and birth weight of 267 children; of them, 222 (83%) were diagnosed with IGHD, 3 (1%) with MPHD, and 42 (16%) with ISS.

**Table 2:** Association between the growth disorder diagnosis, birth presentation, sex and birth weight χ²: Chi-squared test IGHD: Isolated Growth Hormone Deficiency; MPHD: Multiple Pituitary Hormone Deficiency; ISS: Idiopathic Short Stature.

Variable	Diagnosis			χ²	P-value
	IGHD 222(83%)	MPHD 3(1%)	ISS 42(16%)		
Birth Presentation					
Cephalic Vaginal	194(73%)	2(0.7%)	37(14%)	4.57	0.60
Cephalic Cesarean	22(8.2%)	1(0.4%)	4(1.4%)		
Vaginal Breech	1(0.4%)	0(0%)	1(0.4%)		
Cesarean Breech	5(2%)	0(0%)	0(0%)		
Sex					
Female	84(38%)	2(67%)	26(62%)	9.16	0.01
Male	138(62%)	1(33%)	16(38%)		
Birth weight (kg)					
<2.5	29(11%)	1(0.4%)	8(3%)	1.94	0.38
≥2.5	193(72%)	2(0.7%)	34(13%)		

Birth presentation did not differ significantly across diagnostic groups (χ² = 4.57, P = 0.60). Vaginal delivery was the predominant mode of birth in all groups for cephalic presentation, accounting for 194 children (73%) with IGHD, 2 (0.7%) with MPHD, and 37 (14%) with ISS. Cesarean section delivery was the second most common for cephalic presentation, observed in 22 children (8.2%) with IGHD, 1 (0.4%) with MPHD, and 4 (1.4%) with ISS. Breech presentations were uncommon, with only one vaginal breech delivery reported in both the IGHD and ISS groups and five cesarean deliveries for breech presentation occurring exclusively among children with IGHD.

A significant association was observed between sex and diagnosis (χ² = 9.16, P = 0.01). Males predominated among children with IGHD, comprising 138 cases (62%) compared with 84 females (38%). In contrast, females represented a greater proportion of children with ISS, accounting for 26 cases (62%) compared with 16 males (38%). Although MPHD was rare, two of the three affected children (67%) were female.

Birth weight was not significantly associated with diagnostic category (χ² = 1.94, P = 0.38). Most children in all diagnostic groups had a birth weight of at least 2.5 kg, including 193 children (72%) with IGHD, 2 (0.7%) with MPHD, and 34 (13%) with ISS. Low birth weight (<2.5 kg) was relatively uncommon and was observed in 29 children (11%) with IGHD, 1 child (0.4%) with MPHD, and 8 children (3%) with ISS.

Association between diagnosis and growth outcomes

Table 3 presents the association between the diagnosis of growth disorders and growth velocity, growth hormone (GH) stimulation test results, response to GH therapy over a two-year period, and bone maturity assessment according to chronological age among children with isolated growth hormone deficiency (IGHD), multiple pituitary hormone deficiency (MPHD), and idiopathic short stature (ISS).

**Table 3:** Association between the diagnosis, growth velocity, response to GH treatment and bone assessment according to chronological age *: ANOVA Test **: Chi-squared test (χ²) IGHD: Isolated Growth Hormone Deficiency; MPHD: Multiple Pituitary Hormone Deficiency; ISS: Idiopathic Short Stature.

Variable	Diagnosis				
Growth Velocity Per Time	IGHD (n=222)	MPHD (n=3)	ISS (n=42)	Test statistic	P-value
Growth Velocity per year (mean±SD) cm	3.3±1.01	1.7±1.5	3.6±1.1	5.62*	0.006
Growth Velocity after 2 years (mean±SD) cm	5.4±2.3	5.3±1.2	5.4±2.2	0.003*	0.99
Growth hormone peak test (mean±SD) μg/L	5.3±2.3	5.2±4.6	12.9±3.9	147.1*	<0.0001
Response to GH treatment over 2 years					
Did not catch up	121(45.3%)	2(0.7%)	26(9.7%)	45.61**	<0.001
Catch up in 1^st^ year	67(25.1%)	1(0.4%)	11(4.1%)		
Catch up in 2^nd ^year	34(12.7%)	0(0.0%)	5(1.9%)		
Bone assessment according to chronological age					
Normal	113(42.3%)	0(0.0%)	27(10.1%)	6.47**	0.17
Delayed	100(37.5%)	3(45.3%)	14(5.2%)		
Advanced	9(3.4%)	0(0.0%)	1(0.4%)		

Significant differences in baseline growth velocity were observed among the diagnostic groups (F = 5.62, P = 0.006). Children with MPHD exhibited the lowest annual growth velocity (1.7 ± 1.5 cm/year), followed by those with IGHD (3.3 ± 1.01 cm/year), whereas children with ISS demonstrated the highest growth velocity (3.6 ± 1.1 cm/year). These findings indicate that growth impairment was most pronounced among children with MPHD, reflecting the more severe endocrine dysfunction associated with multiple pituitary hormone deficiencies.

After two years of follow-up, growth velocity increased substantially in all groups, and no significant differences were observed between diagnoses (F = 0.003, P = 0.99). The mean growth velocity after two years was 5.4 ± 2.3 cm in the IGHD group, 5.3 ± 1.2 cm in the MPHD group, and 5.4 ± 2.2 cm in the ISS group. This finding suggests that treatment and follow-up resulted in comparable growth outcomes across the three diagnostic categories despite differences in baseline growth rates.

The GH peak stimulation test differed markedly among the diagnostic groups (F = 147.1, P < 0.0001). Children with ISS had significantly higher peak GH concentrations (12.9 ± 3.9 µg/L) than those with IGHD (5.3 ± 2.3 µg/L) and MPHD (5.2 ± 4.6 µg/L). This result is consistent with the underlying pathophysiology of ISS, in which GH secretion is generally preserved, whereas children with IGHD and MPHD exhibit impaired GH secretion.

A highly significant association was also observed between diagnosis and response to GH treatment over the two-year follow-up period (χ² = 45.61, P < 0.001). Overall, failure to achieve catch-up growth was the most common outcome, occurring in 121 children with IGHD (45.3%), 2 children with MPHD (0.7%), and 26 children with ISS (9.7%). Catch-up growth during the first year of treatment was observed in 67 children with IGHD (25.1%), 1 child with MPHD (0.4%), and 11 children with ISS (4.1%). Catch-up growth during the second year occurred in 34 children with IGHD (12.7%) and 5 children with ISS (1.9%), whereas no children with MPHD achieved catch-up growth during the second year. These findings indicate considerable variability in treatment response among diagnostic groups, with a substantial proportion of children failing to attain adequate catch-up growth despite therapy.

Bone maturity assessment according to chronological age showed no statistically significant association with diagnosis (χ² = 6.47, P = 0.17). Normal bone age was observed in 113 children with IGHD (42.3%) and 27 children with ISS (10.1%), whereas delayed bone maturation was present in 100 children with IGHD (37.5%), all three children with MPHD (45.3%), and 14 children with ISS (5.2%). Advanced bone age was uncommon, occurring in only nine children with IGHD (3.4%) and one child with ISS (0.4%). Although delayed bone maturation appeared more frequent among children with MPHD and IGHD, the overall distribution of bone maturity categories did not differ significantly across diagnostic groups.

Overall, the findings demonstrate that the type of growth disorder is significantly associated with baseline growth velocity, GH secretory capacity, and response to GH therapy. Children with MPHD exhibited the most severe impairment in linear growth, while those with ISS had preserved GH secretion and the highest baseline growth velocity. Despite these baseline differences, growth velocities became comparable after two years of treatment, highlighting the potential effectiveness of therapeutic intervention.

Association between magnetic resonance imaging (MRI) findings and diagnosis of growth disorder

Table 4 demonstrates the association between magnetic resonance imaging (MRI) findings and the diagnosis of growth disorders among 267 children, including isolated growth hormone deficiency (IGHD), multiple pituitary hormone deficiency (MPHD), and idiopathic short stature (ISS). The findings demonstrate that most children had either normal MRI appearances or isolated pituitary hypoplasia, while more complex structural abnormalities were relatively uncommon.

**Table 4:** Association between brain and pituitary gland MRI findings and diagnosis of growth disorder MRI: Magnetic Resonance Imaging; IGHD: Isolated Growth Hormone Deficiency; MPHD: Multiple Pituitary Hormone Deficiency; ISS: Idiopathic Short Stature.

MRI Findings	Total (n=267)	Diagnosis of Growth Disorder		
		IGHD (n=222)	MPHD (n=3)	ISS (n=42)
Normal brain and pituitary	174(65.2%)	139(69.9%)	2(1.1%)	33(19.0%)
Normal brain and pituitary hypoplasia	77(28.8%)	68(88.3%)	1(1.3%)	8(10.4%)
Normal brain and pituitary hypoplasia with Ectopic posterior pituitary gland	3(1.1%)	3(100%)	0(0%)	0(0%)
Empty Sella syndrome	3(1.1%)	2(67%)	0(0%)	1(33.0%)
Pituitary hypoplasia, Choroid plexus cyst	2(0.7%)	2(100%)	0(0%)	0(0%)
Normal Pituitary and Chiari 1 Malformation	2(0.7%)	2(100%)	0(0%)	0(0%)
Normal Pituitary left cranial fossa arachnoid cyst	2(0.7%)	2(100%)	0(0%)	0(0%)
Normal Brain & Pituitary and partial corpus callosum agenesis	1(1.01%)	1(100%)	0(0%)	0(0%)
Pituitary hypoplasia and multiple bilateral cerebral cavernoma	1(1.01%)	1(100%)	0(0%)	0(0%)
Small anterior pituitary	1(1.01%)	1(100%)	0(0%)	0(0%)
Double Pituitary Gland stalk	1(1.01%)	1(100%)	0(0%)	0(0%)

Overall, a normal brain and pituitary gland was the most frequent MRI finding, observed in 174 children (65.2%). Among these patients, the majority were diagnosed with IGHD (139 cases, 69.9%), followed by ISS (33 cases, 19.0%), whereas only two cases (1.1%) had MPHD. This finding suggests that a substantial proportion of children with growth disorders, particularly those with IGHD, may not exhibit detectable structural abnormalities on MRI, emphasizing the functional rather than anatomical nature of many growth hormone deficiencies.

The second most common MRI finding was normal brain anatomy with pituitary hypoplasia, identified in 77 children (28.8%). This abnormality was predominantly associated with IGHD, accounting for 68 cases (88.3%), while only one case (1.3%) occurred in MPHD and eight cases (10.4%) in ISS. The strong predominance of IGHD among children with pituitary hypoplasia highlights the importance of reduced pituitary size as a radiological marker suggestive of growth hormone deficiency.

Rare congenital pituitary and midline abnormalities were identified almost exclusively among children with IGHD. Normal brain and pituitary hypoplasia associated with an ectopic posterior pituitary gland was observed in three children (1.1%), all of whom were diagnosed with IGHD (100%). Similarly, pituitary hypoplasia associated with a choroid plexus cyst was identified in two cases (0.7%), both with IGHD. Other uncommon structural abnormalities, including Chiari I malformation, left cranial fossa arachnoid cyst, partial agenesis of the corpus callosum, multiple bilateral cerebral cavernomas, small anterior pituitary gland, and double pituitary stalk, were each observed in one or two patients and occurred exclusively in the IGHD group. These findings support previous evidence that congenital developmental abnormalities involving the hypothalamic-pituitary axis are strongly associated with growth hormone deficiency.

Empty Sella syndrome was identified in three children (1.1%), with two cases (67.0%) occurring in the IGHD group and one case (33.0%) in the ISS group. Although uncommon, this finding may reflect pituitary structural compromise contributing to impaired growth hormone secretion in some patients.

The MPHD group was notably small (n = 3), limiting definitive conclusions regarding MRI patterns. Nevertheless, MRI findings in these patients included either a normal pituitary gland (66.7%) or pituitary hypoplasia (33.3%), suggesting that significant structural abnormalities were not consistently present despite the presence of multiple hormonal deficiencies.

From a clinical perspective, these results indicate that while normal MRI findings are common among children with growth disorders, pituitary hypoplasia is highly associated with IGHD and may serve as an important radiological indicator of growth hormone deficiency. Furthermore, the exclusive occurrence of several congenital pituitary and midline brain abnormalities within the IGHD group suggests a developmental origin in a subset of affected children. In contrast, children with ISS generally demonstrated either normal MRI findings or only occasional nonspecific abnormalities, supporting the concept that ISS is largely characterized by the absence of identifiable hypothalamic-pituitary structural defects. Overall, the findings underscore the value of MRI in identifying anatomical abnormalities associated with growth hormone deficiency and in differentiating IGHD from other causes of short stature.

## Discussion

Growth hormone deficiency (GHD) is an important endocrine disorder in children. The cause and mechanism of GHD may be congenital, acquired, or idiopathic. There is variation in the severity of GH deficiency and duration according to the involvement of other pituitary hormones or its occurrence as part of a complex syndrome, which may be isolated or associated with multiple pituitary hormone deficiency (MPHD) [1-3]. Current evidence indicates that effective evaluation and management of growth disorders require standardized growth assessment, appropriate endocrine investigations, population-specific growth references, and multidisciplinary diagnostic approaches to optimize growth outcomes and integrate treatment programs to facilitate better adjustment in adult life and long-term health [4-6]. However, diagnostic challenges often complicate the distinction between idiopathic short stature (ISS) and GH deficiency, and ISS remains a controversial indication for growth hormone therapy [7].

This study evaluated the associations between diagnostic categories of growth disorders, perinatal characteristics, growth outcomes, and magnetic resonance imaging (MRI) findings among children diagnosed with isolated growth hormone deficiency (IGHD), multiple pituitary hormone deficiency (MPHD), and idiopathic short stature (ISS). The findings provide important insights into the clinical, auxological, and radiological characteristics of these disorders and are consistent with several previous reports.

The predominance of IGHD (83%) observed in the current study is consistent with contemporary evidence indicating that isolated GH deficiency represents the most common endocrine cause of pathological short stature in childhood. Current recommendations emphasize that growth disorders are heterogeneous conditions requiring comprehensive clinical, biochemical, radiological, and genetic assessment to establish an accurate diagnosis and guide management [7]. Furthermore, the diagnostic classification employed in the present study is aligned with international consensus recommendations and clinical practice guidelines that advocate standardized auxological assessment, GH stimulation testing, and long-term monitoring of growth outcomes in children with suspected GH deficiency [4,6].

Although low birth weight was present in a minority of patients in the present study, the predominance of normal birth weight across all diagnostic groups supports the concept that prenatal growth is often preserved in GH-deficient children. Al Shaikh et al. [8] studied the effect of growth hormone treatment in children with ISS, idiopathic GHD, and those born small for gestational age (SGA). They found that height increased significantly in children with GHD after three years of GH therapy compared with children with ISS, whereas body mass index decreased significantly in children with ISS and SGA. In contrast, Cacciari et al. [9] reported that birth weight may influence subsequent growth patterns and responsiveness to GH therapy, with higher birth weights generally associated with better treatment outcomes. However, the absence of a significant relationship between birth weight and diagnostic classification in the current study suggests that birth weight alone has limited utility in distinguishing among IGHD, MPHD, and ISS.

No significant association was observed between birth presentation and diagnosis. Breech presentation was uncommon and occurred predominantly among children with IGHD. Previous studies have suggested a possible association between congenital hypothalamic-pituitary abnormalities and abnormal fetal positioning. Maghnie et al. [10] reported an increased frequency of breech presentation among children with congenital hypopituitarism, proposing that prenatal hypothalamic-pituitary dysfunction may contribute to altered fetal movement and abnormal delivery presentations. Although a similar trend was observed in the current study, the association did not reach statistical significance, likely because of the small number of breech deliveries.

The significant association between sex and diagnosis, characterized by male predominance in IGHD and female predominance in ISS, is consistent with previous epidemiological observations indicating that boys are more frequently referred for evaluation of short stature and are more often diagnosed with GH deficiency. This phenomenon may reflect referral bias, differences in growth expectations, or biological variations in growth regulation, as discussed in current reviews of GH deficiency and disorders of growth hormone deficiency [2].

The growth outcome analysis demonstrated significant differences in baseline growth velocity, with MPHD patients exhibiting the lowest growth rates and ISS patients showing the highest. The markedly lower GH peak concentrations observed among children with IGHD and MPHD compared with ISS further validate the diagnostic classifications and align closely with the consensus diagnostic criteria established by the Growth Hormone Research Society and updated clinical recommendations [4,6].

Despite significant baseline differences, growth velocities became comparable among the three diagnostic groups after two years of treatment and follow-up. These findings support the effectiveness of therapeutic intervention in improving growth outcomes and are consistent with the recommendations of the Drug and Therapeutics and Ethics Committees of the Pediatric Endocrine Society, which emphasized individualized GH treatment and close monitoring to optimize growth response [7]. Similarly, Lee et al. [11] highlighted the importance of early identification and treatment of growth disorders, particularly among children born small for gestational age who fail to demonstrate adequate catch-up growth. The observed improvement in growth velocity across diagnostic groups reinforces the therapeutic benefits of GH treatment when appropriately administered and monitored.

However, a substantial proportion of children failed to achieve catch-up growth despite treatment, particularly among those with IGHD. This variability in treatment response is consistent with previous reports demonstrating that GH responsiveness is influenced by multiple factors, including disease severity, treatment adherence, age at treatment initiation, genetic factors, and baseline auxological characteristics [2,4,6]. The findings therefore support current recommendations advocating individualized treatment strategies and long-term follow-up to optimize outcomes.

Bone age assessment revealed no statistically significant differences among diagnostic groups, although delayed bone maturation was more common in children with MPHD and IGHD. These findings are consistent with the established clinical characteristics of GH deficiency, where delayed skeletal maturation frequently accompanies impaired linear growth [8]. The absence of significant differences may reflect the considerable overlap in bone age findings among different causes of short stature. Cacciari et al. [9] examined final height outcomes in children with isolated idiopathic GH deficiency who were born small, appropriate, or large for gestational age. They concluded that despite similar height deficits and bone age delay at treatment initiation, final height outcomes differed substantially according to birth weight.

The MRI findings observed in the present study strongly support the important role of neuroimaging in the evaluation of childhood growth disorders. These findings align with the conclusion of Alyahyawi, who stated that children with isolated GHD exhibited significant growth impairment and clinical characteristics consistent with the disorder. Neuroradiological abnormalities are common among patients with severe growth hormone deficiency. Therefore, radiological assessment, including MRI of the pituitary gland, is recommended in patients with severe isolated growth hormone deficiency [3]. Moreover, normal MRI findings were present in most children (65.2%); pituitary hypoplasia represented the most common structural abnormality and was predominantly associated with IGHD. These findings closely resemble those reported by Maghnie et al. [10], who demonstrated that pituitary hypoplasia, ectopic posterior pituitary gland, and pituitary stalk abnormalities are among the most frequent MRI abnormalities identified in children with GH deficiency. Lee et al. [11] further stated that bone age is not a reliable predictor of height potential in children born SGA and that a standard evaluation for short stature should be performed, including assessment for GH deficiency when clinically indicated. Maghnie et al. [12] also reported a significant association between anatomical pituitary abnormalities on MRI and functional endocrine abnormalities in children with GHD. Similarly, Jung et al. [13] reported that pituitary hypoplasia and pituitary stalk interruption syndrome (PSIS) were the most common MRI abnormalities among children with GHD, supporting the routine use of MRI in the diagnostic evaluation of these patients.

The identification of ectopic posterior pituitary gland and other congenital hypothalamic-pituitary abnormalities exclusively among children with IGHD further supports previous evidence linking developmental abnormalities of the hypothalamic-pituitary axis with GH deficiency. Reynaud et al. [14] described pituitary stalk interruption syndrome (PSIS) as a rare congenital disorder of the hypothalamic-pituitary region and an important structural cause of GHD. PSIS is characterized by anterior pituitary hypoplasia, ectopic posterior pituitary, and an absent or interrupted pituitary stalk and may present as either GHD or MPHD. Likewise, Maghnie et al. [15] investigated hypothalamic-pituitary dysfunction in GH-deficient patients with pituitary abnormalities and concluded that most patients with IGHD or MPHD likely have a primary hypothalamic releasing hormone deficiency, even when pituitary hypoplasia is present. They further suggested that MRI has diagnostic and prognostic value in differentiating patients at risk of developing MPHD from those likely to maintain isolated GHD.

The predominance of normal MRI findings among children with IGHD in the present study is also consistent with previous literature demonstrating that many cases of GH deficiency are functional rather than structural. MRI abnormalities are valuable when present but are not required for diagnosis because a substantial proportion of children with confirmed GHD have normal neuroimaging findings. Similarly, Maghnie et al. [10] reported MRI abnormalities in only approximately one-quarter of children with non-acquired GHD, indicating that normal imaging findings remain common.

The rarity of significant MRI abnormalities among children with ISS observed in the present study further supports the concept that ISS is primarily characterized by the absence of identifiable structural hypothalamic-pituitary defects. This observation is consistent with the findings of Sisley et al. [16], who demonstrated that pathological causes of short stature are relatively uncommon among children evaluated for growth concerns and that many children ultimately have normal growth variants or idiopathic etiologies. The predominance of normal MRI findings among ISS patients therefore reinforces the role of MRI in differentiating endocrine and structural causes of short stature from idiopathic conditions.

Although the MPHD group in the current study was very small, the observation that affected children exhibited severe growth impairment and delayed bone maturation is consistent with findings reported by Mamilly et al. [17], who demonstrated that structural MRI abnormalities and greater hormonal deficits are more frequently encountered among children with severe GHD and MPHD. The limited number of MPHD cases, however, precludes definitive conclusions regarding MRI patterns in this subgroup.

The use of growth assessment standards and reference data is fundamental to the identification of short stature and growth disorders. The present study employed internationally accepted growth criteria consistent with the WHO Child Growth Standards, which define short stature as height below two standard deviations from age- and sex-specific norms. In addition, the findings are particularly relevant within the Saudi population because they can be interpreted using nationally developed growth references that provide population-specific standards for assessing growth among Saudi children and adolescents [5,18].

Overall, the findings of the present study support current international recommendations emphasizing comprehensive assessment of children with growth disorders through auxological evaluation, GH stimulation testing, bone age assessment, and MRI examination when indicated. Moreover, this study demonstrates that MRI is particularly valuable for identifying congenital hypothalamic-pituitary abnormalities associated with IGHD while also confirming that many affected children may exhibit normal neuroimaging findings. Furthermore, the improvement in growth velocity following treatment highlights the potential benefits of early diagnosis and appropriate therapeutic intervention in optimizing growth outcomes.

Study limitations

This study has four main limitations: (1) The diagnostic groups were highly imbalanced, with IGHD comprising most cases and only three patients diagnosed with MPHD, limiting the reliability of comparisons involving MPHD. (2) The retrospective single-center design may introduce selection bias and restrict the generalizability of the findings. (3) Several important confounding factors, including genetic, nutritional, socioeconomic, pubertal, and treatment-related variables, were not assessed. (4) MRI evaluation was limited to conventional structural imaging, without advanced neuroimaging or genetic investigations, potentially overlooking subtle hypothalamic-pituitary abnormalities.

Overall, despite providing valuable insights into the clinical, radiological, and treatment characteristics of pediatric growth disorders, the findings should be interpreted cautiously. Larger multicenter prospective studies incorporating comprehensive genetic and endocrine assessments are needed to validate and extend these results.

## Conclusions

Growth disorder among children represents a significant clinical and psychosocial concern requiring systematic evaluation to identify underlying etiologies, particularly treatable endocrine causes such as growth hormone deficiency. This study demonstrates that isolated growth hormone deficiency (IGHD) is the predominant cause of growth disorders among children, accounting for more than four-fifths of all diagnoses. Males were more frequently affected than females, although females tended to present and receive a diagnosis at a significantly younger age, suggesting earlier clinical recognition and referral. Perinatal factors, including birth presentation and birth weight, showed no significant association with growth disorders, indicating that these conditions are unlikely to be attributable to adverse birth-related events. Children with different diagnostic categories exhibited distinct baseline growth characteristics. Multiple pituitary hormone deficiency (MPHD) was associated with the most severe impairment in growth velocity, followed by IGHD and idiopathic short stature (ISS). Despite these initial differences, growth hormone therapy resulted in comparable growth velocities across diagnostic groups after two years, highlighting the effectiveness of treatment in restoring linear growth regardless of the underlying diagnosis. MRI evaluation revealed that normal hypothalamic-pituitary anatomy was the most common radiological finding, even among children with confirmed IGHD. This observation indicates that structural abnormalities are not required for the development of growth hormone deficiency and supports the concept that many cases of IGHD are primarily functional rather than anatomical in origin. Nevertheless, MRI remains valuable for identifying the minority of patients with pituitary hypoplasia or other congenital hypothalamic-pituitary abnormalities that may contribute to growth failure.

Overall, the findings suggest that growth disorders in children are predominantly characterized by IGHD, are influenced more by endocrine dysfunction than by perinatal factors or overt structural brain abnormalities, and respond favorably to growth hormone therapy, which effectively normalizes growth velocity over time. These results emphasize the importance of early diagnosis, comprehensive endocrine assessment, and targeted imaging evaluation to optimize clinical outcomes in children with impaired growth.
